# From Cheese-Making to Consumption: Exploring the Microbial Safety of Cheeses through Predictive Microbiology Models

**DOI:** 10.3390/foods10020355

**Published:** 2021-02-07

**Authors:** Arícia Possas, Olga María Bonilla-Luque, Antonio Valero

**Affiliations:** Department of Food Science and Technology, University of Córdoba, Agri-Food Campus of International Excellence ceiA3, Campus Rabanales s/n, Crta. Madrid-Cádiz km 396A, 14014 Córdoba, Spain; v32boluo@uco.es (O.M.B.-L.); bt2vadia@uco.es (A.V.)

**Keywords:** fermented foods, food safety, predictive mathematical modeling, foodborne pathogens, dairy products, outbreaks

## Abstract

Cheeses are traditional products widely consumed throughout the world that have been frequently implicated in foodborne outbreaks. Predictive microbiology models are relevant tools to estimate microbial behavior in these products. The objective of this study was to conduct a review on the available modeling approaches developed in cheeses, and to identify the main microbial targets of concern and the factors affecting microbial behavior in these products. *Listeria monocytogenes* has been identified as the main hazard evaluated in modelling studies. The pH, a_w_, lactic acid concentration and temperature have been the main factors contemplated as independent variables in models. Other aspects such as the use of raw or pasteurized milk, starter cultures, and factors inherent to the contaminating pathogen have also been evaluated. In general, depending on the production process, storage conditions, and physicochemical characteristics, microorganisms can grow or die-off in cheeses. The classical two-step modeling has been the most common approach performed to develop predictive models. Other modeling approaches, including microbial interaction, growth boundary, response surface methodology, and neural networks, have also been performed. Validated models have been integrated into user-friendly software tools to be used to obtain estimates of microbial behavior in a quick and easy manner. Future studies should investigate the fate of other target bacterial pathogens, such as spore-forming bacteria, and the dynamic character of the production process of cheeses, among other aspects. The information compiled in this study helps to deepen the knowledge on the predictive microbiology field in the context of cheese production and storage.

## 1. Introduction

Cheeses are milk-based foods widely produced and consumed throughout the world [1]. The large acceptability of cheeses by consumers can be attributed to their pleasant sensorial characteristics, good nutritional properties, versatility and by the advent of innovative products with novel ingredients, packaging, and sale formats [1,2]. The organoleptic characteristics of a specific type of cheese, including texture, aroma, and flavor, are dependent on their constituent compounds and molecules, e.g., volatile compounds, free amino acids, phenols, etc., which in turn are influenced by the milk origin, the endogenous microbiota of milk, starter cultures used for its elaboration, the microbial populations present in production environments, and technological aspects of the production processes [1].

During cheese-making, coagulation of milk proteins takes place by means of the addition of rennet or other coagulating agents, resulting in curd formation, which is followed by whey draining [3]. Cheeses are classified according to firmness, from “soft” to “extra hard”, and according to principal ripening from “ripened” to “in brine” [3]. Ripened cheeses are held under controlled conditions for varying periods of time, to develop their biochemical, physicochemical, and organoleptic characteristics [3,4]. Moreover, processed cheeses are products that result from cheese heating and blending with melting salts and other dairy and non-dairy ingredients [5,6].

The transmission of bacterial pathogens during cheese-making, ripening, and storage can be attributed to direct contamination or cross-contamination events during processing, in retail and domestic environments [7,8,9]. While raw milk is considered the primary source of contamination of cheeses [10], the ability of pathogens to form biofilms and persist on food contact surfaces has been related to cross-contamination during production [9,11]. The hands of workers and cheese-contact surfaces have been identified as vehicles for *Staphylococcus aureus* in processing plants [12,13]. Hotspot contaminations have previously been observed at the interface between reception of raw materials and industrial processing areas, while for some pathogens such as *Listeria monocytogenes*, contamination could also be disseminated in the entire processing plant, including floor drains [14,15]. Moreover, unsafe handling and inadequate storage practices in domestic environments have been shown to contribute to cheese contamination and to favor microbial growth or persistence, increasing the risk of illness associated with cheese consumption [9].

The relatively low pH, due to the presence of organic acids (mainly lactic, acetic, butyric, or sorbic acid), and the low moisture content of some types of cheeses reduce the growth and survival capacity of pathogenic bacteria during shelf-life [16,17]. Low refrigeration temperatures, elaboration with pasteurized milk, and the use of bio-protective cultures represent additional hurdles to avoid microbial proliferation and persistence in these products [16,17]. Despite that, in recent decades cheeses have been linked to many outbreaks of illnesses in Europe and elsewhere in the world [9,18]. Soft cheeses elaborated with raw milk have been the most common vehicle of foodborne pathogens, although pasteurized-milk cheeses have also been carriers of the causing agents in outbreaks [19,20,21]. In European countries, cheese production and sale are subjected to Regulation (EC) No. 2073/2005 and microbiological criteria vary according to the microorganism of concern [22]. To support the compliance with current microbiological criteria, it is crucial to evaluate and understand the influence of extrinsic and intrinsic factors that affect microbial behavior in these ready-to-eat (RTE) foods.

Predictive models are mathematical equations to quantitatively describe microbial responses in foods [23]. The use of challenge testing and predictive microbiology models have been recognized as relevant tools for food operators to verify if microbial levels in cheeses might exceed the established microbiological criteria and to evaluate the impact of control measures and the consequences of cross-contamination events during production, distribution, and shelf-life [8,24]. In this sense, these models may assist in the development of Hazard Analysis and Critical Control Points (HACCP) plans, in the improvement of Food Safety Objectives, and in Quantitative Microbial Risk Assessments (QMRA) [25,26]. To date, predictive models have been applied to describe the fate of various pathogenic bacteria such as *L. monocytogenes* [27,28,29], non-enterohemorrhagic *Escherichia coli* [30], and *S. aureus* [31,32] in QMRAs concerning the consumption of cheese.

The aim of this review, therefore, was to gather information on the mathematical predictive models that have been developed in the context of cheese production and storage. An overview of the most important factors governing microbial behavior in cheeses that have been considered in modeling studies is also provided. Finally, this review also provides information on the main bacterial pathogens of concern in cheeses considered for the performance of fate studies.

## 2. Target Bacterial Pathogens in Fate Studies

It is well known that many cheese types have physicochemical characteristics and shelf-life duration compatible with the growth or survival of pathogenic bacteria [33,34]. Moreover, there is abundant information in the scientific literature dealing with challenge test or fate studies of pathogens in different cheese-making, ripening, or storage conditions [35,36]. Selection of target microbial pathogens for fate studies may be based on their representativeness in the food matrix under evaluation and their potential to produce a public health risk after being ingested.

As *L. monocytogenes* is ubiquitous and psychrotrophic, the production of cheeses free of this pathogen is a challenge for producers [28,37]. As a result, most outbreaks linked to the consumption of cheeses have been caused by *L. monocytogenes* [38,39,40,41]. The European Regulation (EC) No. 2073/2005 establishes a tolerance of a maximum of 100 cfu/g of *L. monocytogenes* in RTE foods, including cheese, if food operators can demonstrate that these products do not allow growth of the pathogen during shelf-life [22]. Otherwise, the absence of *L. monocytogenes* in 25 g of product is required. This regulation also mentions that products with pH ≤ 4.4 or a_w_ ≤ 0.92 and with pH ≤ 5.0 and a_w_ ≤ 0.94 are considered unable to support *L. monocytogenes* growth [22].

Many salmonellosis outbreaks linked to the consumption of cheeses have been reported worldwide in recent years [42,43,44,45,46,47], which evidences that *Salmonella* is also a pathogen of concern in these food matrices. According to European Regulation No. 2073/2005, the current criterion for *Salmonella* spp. in cheeses is “absence (non-detection) of the pathogen in 25 g of product” (*n* = 5, c = 0) for products placed on the market during their shelf-life [22].

*S. aureus* is one of the main pathogens found in cheeses produced with raw milk and is a common cause of bovine mastitis, being detected in bulk tank milk at high prevalence rates [48,49]. Since enterotoxin-producing *S. aureus* exhibits a high osmotolerance, it can grow or survive in cheeses with a_w_ levels as low as 0.86 [50]. Pasteurization inactivates *S. aureus* in milk, but not their previously synthesized enterotoxins, which can remain in cheeses and cause food poisoning when ingested at low doses [51,52]. Production of toxins has been reported when the microorganism is present in foods at approximately 10^5^ cfu/g or higher numbers [53,54]. Current regulations in the European Union (EU) establish different criteria concerning the presence of coagulase-positive staphylococci during the manufacturing process according to the cheese type and depending on whether the milk is submitted to heat-treatments prior to cheese elaboration [22]. Overall, if concentrations of coagulase-positive staphylococci detected in samples are higher than 10^5^ cfu/g, the batches might be tested for staphylococcal enterotoxins, which may not be detected in 25-g cheese samples during the product’s shelf-life [22].

Enterohemorrhagic *E. coli* can cause disease at levels lower than 10 cfu/g and are able to grow at the relatively low pH (4.0–4.5) characteristic of some fermented foods [55,56]. Various outbreaks associated with the consumption of raw-milk cheeses caused by Shiga toxin-producing *E. coli* (STEC) have been documented worldwide in the last decade [57,58,59,60]. The *E. coli* O157:H7 strain implicated in one outbreak, related to the consumption of Gouda cheese in Canada in 2013, was isolated in one core sample obtained from an intact cheese wheel 83 days after the beginning of production [58]. This outbreak has called into question the efficacy of ripening for more than 60 days as an alternative to pasteurization to preclude the presence of pathogenic bacteria in cheeses elaborated with raw milk. Moreover, cheese was the most reported incriminated food in outbreaks of strong evidence caused by STEC in the EU in 2018 [61].

Due to the above-mentioned, *L. monocytogenes*, *Salmonella* spp., *S. aureus*, and STEC are the main target foodborne pathogens evaluated in fate studies performed with cheeses. The behavior of *Yersinia enterocolitica* has also been evaluated [62] and surveys have indicated that this psychrotrophic pathogen can overcome the manufacturing process of cheeses and be present in the final products [63]. Although less frequently associated with outbreaks, *Campylobacter* [64] has also been implicated in cases of illness transmitted by cheeses and the evaluation of its behavior in these food matrices would be worthwhile.

*Bacillus* spp. and *Clostridium* spp. are the main genera of spore-forming bacteria found in processed cheeses [6]. *Clostridium botulinum* caused an outbreak associated with a dessert made with mascarpone cheese [65]. Both vegetative cells and spores of *Clostridium sporogenes* were more resistant than *E. coli* O157:H7, *Salmonella* spp., *L. innocua*, and *S. aureus* in a vacuum-packed canned pasteurized-milk cheese [66]. Hence, fate studies to evaluate the responses of spore-forming bacteria to different formulations, packaging and storage conditions are crucial to increasing the stability and safety of cheeses.

## 3. Main Factors Governing Microbial Fate in Cheeses

Fate studies performed in cheeses have indicated that microbial behavior in these matrices is directly or indirectly governed by many intrinsic, extrinsic, or implicit factors during production and storage. In general, the most common factors evaluated as independent/explanatory variables in modeling studies are the physicochemical characteristics of cheeses, i.e., a_w_/NaCl and the pH, the undissociated lactic acid concentration and temperature [24,26]. These factors and their interactions condition the microbial behavior of microbial hazards in cheeses, which can grow or die-off during the production processes and/or storage. In addition, other aspects, which interfere in the characteristics and safety of cheeses, have been evaluated in modeling studies; these include the impact of the application of raw or pasteurized milk for cheese-making, the addition of starter cultures for cheese elaboration and factors inherent to the contaminating pathogen [8,67]. It is important to highlight that most of the fate studies performed with cheese had focused on the behavior of *L. monocytogenes* and, therefore, further research is needed to elucidate/confirm the effects of factors on other foodborne pathogens.

### 3.1. Physicochemical Characteristics: a_w_ and pH

Changes in physicochemical characteristics during the production processes of cheeses play an important role on microbial behavior. The shift from pathogen growth to survival may be observed if the a_w_ and pH values are reduced to values lower than the minimum requirements for pathogen growth. Thus, it is important to know beforehand the pH and a_w_ combinations that allow bacterial growth; these are reported in many studies and guidelines [68].

When comparing different types of cheeses, the higher the a_w_ the higher is the microbial growth capacity. The growth potential of *L. monocytogenes* in soft and semi-soft cheeses was noted to be substantially higher than in semi-hard cheeses, which present lower a_w_ [34]. On the other hand, regarding a specific type of cheese, the decrease of a_w_ during ripening and/or storage reduces the microbial growth capacity. Wemmenhove et al. [69] indicated that low a_w_, as presented after prolonged ripening times, led to the complete growth inhibition of *L. monocytogenes* in Gouda cheese. In addition to ripening times, a_w_ of cheeses is dependent on the position within a cheese, i.e., core and rind. Smeared cheeses elaborated with pasteurized milk showed a decrease in numbers of *L. monocytogenes* during ripening which was more marked on the rind (minimum a_w_ value = 0.79) than in the core (minimum a_w_ value = 0.815) [26]. In agreement, increased outgrowth of the pathogen was noted when inoculation was performed in the core rather than in the rind of soft cheeses [34]. The lowering of a_w_ on the rind is attributed to the pronounced loss of moisture during ripening of cheeses, which leads to the increase in salt concentration in this part of the cheese [26].

The pH of cheeses decreases with the production of lactic acid during fermentation of lactose. *L. monocytogenes* showed a lag phase when inoculated in a young acidic cheese (13 days of maturation), starting to grow at days 22–23 of ripening, once an increase in pH values was noted. When inoculation was performed in more mature cheeses, with higher pH, growth started immediately after inoculation [24]. With regard to pH evolution over time, in smeared or mold-ripened cheeses, after a first decrease during cheese-making and at the early stages of ripening, the pH rises to levels around neutral during ripening as a result of the metabolism of lactate by the microbial mixture present in the smearing solution and/or yeasts and molds, which results in the formation of alkaline metabolites, such as ammonia [24,69,70,71]. Ferrier et al. [71] also observed that the pH of a smeared soft cheese increases from the core to the rind, which indicates more intense deacidification activity in the rind compared to the core. Overall, these findings indicate that the lower the pH, the lower the growth/survival potential of pathogenic bacteria in cheeses [24], either due to the direct effect of acidity on microbial cells or due to the increase in the degree of dissociation of organic acids [69].

### 3.2. Lactic Acid

Lactic acid is produced due to lactose fermentation by the endogenous microbiota of milk or the starter cultures added during cheese formulation. Lactic acid is active against microorganisms in its undissociated form. The concentration of undissociated lactic acid in cheeses increases with the increase in total lactic acid content and is negatively correlated with pH. In addition, it decreases during the ripening process, due to lactate metabolization [71]. In addition to lowering the pH, undissociated lactic acid permeabilizes the membranes of bacteria and potentiates the effects of other antimicrobial substances [72].

*L. monocytogenes* presented lower growth capacity at higher undissociated lactic acid concentrations in cheeses [24,26,73]. Undissociated lactic acid was reported to be the main factor inhibiting growth of *L. monocytogenes* in Gouda cheese, besides pH, a_w_ and temperature [69]. These authors observed that if the concentration of undissociated lactic acid is insufficient to ensure complete growth inhibition, the other mentioned growth-inhibiting factors become more important [69]. Thus, findings of previous investigations have indicated that when evaluating the behavior of microbial pathogens in cheeses for the development of predictive models and/or to define their growth-no growth boundaries, undissociated lactic acid must be included as a factor, in addition to temperature, a_w_, and pH.

### 3.3. Storage Temperature

Among the environmental conditions affecting microbial behavior during storage, temperature is the most widely investigated [74,75]. It has been demonstrated that when the physicochemical characteristics of cheeses allow pathogen growth, growth rates are higher at higher storage temperatures [8,24,76].

Growth rates of *L. monocytogenes* on a sliced vacuum-packed Hispanic-style fresh cheese were higher during storage at 10 °C than at 4 °C for 35 d [76]. In addition, *L. monocytogenes* showed higher growth potential in different types of cheeses stored at 14 °C than at 7 °C [34]. The information derived from fate studies indicates that when the physicochemical characteristics of a cheese allow pathogen growth, proper storage temperature alone is not sufficient to inhibit growth [77,78]. Therefore, further studies are needed to develop and optimize formulations, biopreservation strategies, or to evaluate interventions, such as high hydrostatic pressure processing application to better control the growth of pathogens in these products [16].

On the other hand, when products do not allow pathogen growth, reductions during cheese storage have been shown to be temperature-dependent, with bacterial cells surviving for a longer period at lower temperatures [79,80,81]. For instance, the time needed for a 3-log reduction of *L. monocytogenes* was nearly 8.7 days at 12 °C and 5.5 days at 17 °C [79]. Furthermore, *L. monocytogenes* inactivation rates in a processed cheese increased with the increase of storage temperature from 4 to 22 °C [82]. This issue is attributed to the fact that in low-pH environments like some types of cheeses, the metabolism of bacterial cells is very intense to overcome the acidic stress and maintain the vital processes, which results in the exhaustion of their energy resources. The consumption of resources is accelerated at temperatures which are closer to the optimum temperatures for growth. Since cells are not so active at low temperatures, the exhaustion of energy resources is delayed and cells can survive for longer periods [79,83].

From the microbiological safety point of view, if cheeses’ physicochemical characteristics do not allow the growth of pathogenic microorganisms, it would be more appropriate to store them at ambient temperatures than at refrigeration temperatures, since at ambient temperatures inactivation rates would be higher [84]. On the other hand, those cheeses allowing microbial growth may be stored at lower temperatures.

### 3.4. Raw Milk vs. Pasteurized Milk

Raw-milk cheeses have been the most common vehicle of foodborne illnesses in comparison with pasteurized-milk cheeses [1], and this has motivated the performance of many fate studies to evaluate or/and compare microbial behavior in products elaborated with non-heated and heated milk. Microbial growth or survival rates estimated in cheeses have been shown to be dependent on whether milk is submitted to heat treatments prior to production. It is worth mentioning that in these studies, the pathogen is artificially inoculated in raw or pasteurized milk to evaluate its behavior during cheese-making and storage, simulating insufficient heat treatments of milk or cross-contamination events in processing environments.

Pasteurized milk favored the growth of *L. monocytogenes* when compared with raw milk during cheese-making of a smeared cheese, with an increase of pathogen levels of 2.02 log-units in the former while no increase was observed in the latter [26]. Semi-hard Minas cheese produced with pasteurized milk was found to favor the growth of *L. monocytogenes* when compared with raw-milk cheese during non-starter assisted cheese-making and ripening [67]. The same *L. monocytogenes* behavior was observed in soft Minas cheese elaborated without starter cultures during cheese-making followed by 15 days of storage [35]. This was also demonstrated by Tiwari et al. [8] who found out that *L. monocytogenes* grew at a slower rate on a semi-soft raw-milk cheese (0.05–0.37 d^−1^) when compared with pasteurized cheese milk (0.18–0.85 d^−1^) over 28 days of storage.

When a reduction on pathogen levels rather than growth was noted on cheeses, it was reported that inactivation rates were higher in raw-milk cheeses than in pasteurized cheeses. For instance, Campagnollo et al. [67] found out that shorter times were required to attain 4 log-reductions of *L. monocytogenes* in semi-hard Minas cheeses containing added Lactic Acid Bacteria (LAB) produced with raw-milk, i.e., 15 days, compared with pasteurized-milk cheese, i.e., 21 days. It was also shown, *L. monocytogenes* inactivation was faster in raw milk (−0.0260 h^−1^) than in pasteurized (−0.0182 h^−1^) semi-hard Minas cheese during ripening [85].

The above-mentioned comparisons concern fate studies in which the only difference between the elaboration process is the use of raw or pasteurized milk. The differences in growth or survival potential noted in these fate studies may be partially explained by bacterial competition between the evaluated pathogen and the background microbiota of cheeses. Due to the elimination of the background microbiota during milk pasteurization, bacterial competition is expected to be higher in raw-milk cheeses, which can explain the lower growth rates or higher inactivation rates of microbial pathogens in raw-milk cheeses [34]. In addition, the lactoperoxidase enzyme present in raw milk, which can exert bacteriostatic activity against many pathogenic bacteria, is denatured by heat treatments and therefore is not active in pasteurized cheeses [8,86].

### 3.5. Starter Cultures and Their Bacteriocins

Starter cultures composed of LAB strains have been frequently used in the manufacture of cheeses, as they contribute to the development of the organoleptic properties of these products [87]. Campagnollo et al. [67] isolated LAB strains from samples of artisanal Minas cheeses and after confirming their anti-listerial capacity in vitro at 7 and 37 °C, strains were selected for further cheese production. A pool of six strains of LAB was used at a concentration of 10^6^–10^7^ cfu/mL of milk. *L. monocytogenes* exhibited lower growth potential in raw and pasteurized soft Minas cheeses in the presence of LAB. In raw or pasteurized semi-hard cheeses, *L. monocytogenes* could grow in the absence of LAB while a survival pattern was noted in the presence of LAB, yielding more than 4-log reductions of the pathogen [67]. The anti-listerial effects of LAB strains were further investigated and confirmed in Minas fresh [35] and semi-hard [85] cheeses.

Isolation of LAB strains with antimicrobial activity against pathogens is common, as demonstrated for *S. aureus* in milk [88], *L. monocytogenes* [89,90], *Bacillus cereus*, and *Clostridium perfringens* [91] in cheeses. In general, the presence of LAB accelerates pH decline in cheeses during fermentation, which negatively affects the growth potential of microbial pathogens. The mechanisms by which LAB suppress the growth of microbial pathogens vary among strains but have been associated with competition for nutrients and production of antimicrobial compounds, including bacteriocins [67,92]. The extent of competition and production of bacteriocins by LAB have been shown to be influenced by a series of factors, which include temperature and pH [92].

The antimicrobial effects of adding purified LAB-bacteriocins, mainly nisin, against Gram-negative and Gram-positive bacteria in cheeses have been extensively reported [93,94,95]. The mechanisms of action of bacteriocins include the increase in permeability of the membranes of microbial cells, weakening of cell walls, collapse of proton motive force, which lead to the leakage of intracellular substances and result in bacterial inactivation [92,93]. Increasing nisin residual concentrations from 0.56 to 5.28 ppm extended *L. monocytogenes* lag times in processed cheeses, at temperatures lower than 15 °C [96]. The antimicrobial efficacy of nisin has been shown to be higher at higher pH values and at lower temperatures [95,96]. Moreover, increasing nisin concentrations from 0 to 240 ppm, reduced the potassium sorbate and sodium chloride (NaCl) concentrations required to achieve the same probability of growth of *C. sporogenes* in a processed cheese analogue [97]. Hence, the use of bacteriocins such as nisin could contribute to the development of low-salt and healthier formulations of cheeses and to the optimization of processing conditions without compromising the microbiological safety of these RTE foods.

### 3.6. Factors Inherent to the Target Pathogen

The stress responses of bacterial cells when exposed to the acid conditions encountered in cheeses, including production of stress-related proteins, may be an issue during production and storage. Microbial stress responses can lead to adaptation or cross-protection mechanisms, which can increase the survival/growth capacity of cells or enable the recovery of injured cells back to their healthy status [79]. Although no differences between the survival rates of non-acid and acid-adapted cells were revealed when accessing the survival of *L. monocytogenes* in a traditional Greek cheese in the study by Mataragas et al. [79], higher survival capacity of acid-adapted *L. monocytogenes* and *E. coli* O157:H7 compared with non-acid adapted strains was observed on fresh cheeses [98].

Overall, the physiological state of cells may determine that some different strains of the same microorganism exhibit better growth/survival capacity than others during cheese production and shelf-life [24]. Survival and growth of *L. monocytogenes* was reported to be strain-dependent in Katiki, a traditional Greek soft cheese [99]. In support of this finding, other authors also found significant variation in the growth and inactivation rates of different strains of *L. monocytogenes* in cheeses [24,36]. These differences are relevant when selecting strains for challenge tests and risk assessments. For instance, the *L. monocytogenes* strain Scott A is frequently selected for use in challenge tests together with other strains as it has shown to be resistant and able to survive for prolonged periods of time in contaminated dairy products [80,81,82,100]. To account for this inter-strain variability that can occur in contaminated milk and cheese processing environments, the application of a cocktail composed of different strains is recommended when performing challenge tests and has been reported in many studies [36,81,100].

### 3.7. Organic Acids and Salts

The effects of organic acids rather than lactic acid have been evaluated as independent variables in modeling studies [101]. Inactivation of *L. monocytogenes* increases by increasing the sorbic acid concentration from 0 to 1000 ppm in the water phase in Cottage cheese [73]. Increasing concentrations of acetic (568–3483 ppm) and citric (518–38,282 ppm) acids have also been shown to reduce the growth potential of the pathogen on processed cheese [5,96]. The mechanisms of growth suppression by these acids seem to be similar to those of lactic acid. Nevertheless, not much information is published on the antimicrobial effects of such acids in cheese matrices and, therefore more studies are needed to elucidate their joint action with other physicochemical and environmental factors.

Melting salts, including phosphates, are well known to contribute to the microbiological safety of spreadable processed cheese [5,102]. By increasing the pH of a spreadable cheese to values higher than 5.7 and the moisture content to values higher than 54%, it was necessary to increase both NaCl and/or phosphates concentrations to avoid *C. botulinum* toxin production [102]. This study highlights the important role of salts against spore-forming bacteria. Moreover, the increase of phosphates salt concentrations reduced the growth of *L. monocytogenes* in a spreadable processed cheese [5,96]. The presence of salts reduces the a_w_ of cheeses, which, therefore, may not be considered as an isolated entity. In general, the higher the concentration of salts, the higher the microbiological safety of cheeses.

## 4. Models of Microbial Behavior during Cheese Production and Storage

Many models of microbial behavior in cheeses during the production process (cheese-making and ripening) and storage have been developed. Depending on the target microorganism requirements for growth, the characteristics of the cheeses and environmental conditions, either a growth or a survival pattern of microbial levels have been noted. In other cases, the objective of the study was to define the microbial interface of growth-no growth in the so-called growth boundary models. Primary models have been used to estimate changes in pathogen levels over time and are based on the actual observed kinetic data, while secondary models are used to quantify the effect of extrinsic or intrinsic factors of the foods on pathogen survival, i.e., inactivation/survival rates, or growth kinetic parameters, i.e., lag times and growth rates [23]. Primary and secondary models can be coupled, which is defined in this study as tertiary modeling, so it is possible to estimate changes in bacterial concentration along with cheese production and storage as a function of environmental and intrinsic factors [26].

Although fitting a primary model followed by fitting a secondary model using the parameters estimated through primary modeling, i.e., two-step modeling approach, has been extensively applied in the context of cheese production and storage, different approaches have also been performed and will be discussed in the following sections. A comparison between the one-step procedure with the classical two-step procedure was performed by Martinez-Rios et al. [96]. In the one-step procedure the parameters of both primary and secondary models are estimated through one global regression. Most of the fate studies for model development has been performed in cheeses elaborated with cow’s milk, except for some investigations where ewes [84], goat [103], or both ewes and goat [79,81] milk cheeses were evaluated.

### 4.1. Growth Models

Microbial growth models developed in cheese are depicted in Table 1. The Baranyi and logistic primary models have been the most applied to fit microbial growth data over time observed during the production process of cheeses [26,67,70,104]. These models together with the Gompertz model have been also used to describe growth kinetics during storage of cheeses at constant temperatures (Table 1).

Regarding secondary models, some authors reported the presence of a lag phase on growth curves during cheese storage and related lag times to storage temperatures by means of the Ratkowsky [62], hyperbolic [77], exponential [78] and linear [33] models. Moreover, to relate the growth rates with the most important factors governing microbial behavior in cheeses, the cardinal secondary model [5,24,26,73], the Ratkowsky [33,62,77,78] and polynomial models [74,105] have been used.

Several authors highlight that the application of secondary models belonging to the “Cardinal model family” has a large advantage over the others, as they include cardinal parameters with biological significance for the environmental factors considered, such as the minimum, maximum and optimum values of temperature (*T_min_*, *T_max_* and *T_opt_*) for growth, which personalizes the model to the specific microorganism under evaluation [70]. In these models, the maximum growth rate is expressed as a multiplicative effect between the optimum growth rate (*µ_opt_*) and the functions of the different physicochemical parameters and environmental factors, i.e., gamma concept [108]. Furthermore, cardinal models can account for the influence of many individual factors and their interactions, which is an advantage considering that many factors can simultaneously influence microbial behavior in such complex matrices. Finally, these models can be easily extended to account for the effect of other factors on microbial growth [5,96].

### 4.2. Survival Models

Information on the microbial survival models developed in cheese is presented in Table 2. Regarding survival kinetics, some authors have found that the drop in pathogen concentrations followed a period in which pathogen populations remained relatively unchanged, i.e., shoulder effect [80,81]. Once population levels begin to drop, survival kinetics in cheeses generally consists of two phases [79,81]. At first, a fraction of the population is rapidly inactivated and afterwards a slowing down in inactivation, which is known as the tailing effect, is noted. These non-linear survival behaviors have been described by the Geeraerd [79], Gompertz [81], and Weibull [75,80,82,84,109] primary models. The shoulder effect describes the existence of a lag period prior to inactivation representing the initial resistance of microorganisms. The tailing effect has been associated with the development of defensive mechanisms by bacterial cells to survive in harsh environment conditions, e.g., acid stress response during cheese ripening and storage, or to changes in processing parameters such as ripening temperatures and relative humidity which also influences survival rates [79,81]. Another hypothesis to explain differences in survival rates is the heterogeneity within the inoculated bacterial population in terms of resistance [79,81] or the existence of interaction phenomena with the cheese microbiota during fermentation and ripening.

Finally, the Arrhenius [75,82,84] and polynomial [79,81] models have been the most common secondary models used to relate survival kinetic parameters with cheese storage temperatures (Table 2). While the Arrhenius equation was developed for physical chemistry applications, considering the rate of chemical reactions and energy of activation, polynomial models are purely statistical. In this study we encourage the development of more mechanistic models to describe survival/inactivation rates in cheeses as a function of environmental factors, such as the one published by Coroller et al. [110], which extended the gamma concept to study the non-thermal inactivation of *Salmonella* in dried sausages. 

### 4.3. Growth Boundary Models

The aim of applying growth boundary models is to define combinations of physicochemical characteristics or/and environmental conditions that produce growth or inactivation of target pathogens or enable toxin production in cheeses. Binary logistic regression has been used to develop a model to estimate the probability of growth of *L. monocytogenes* in a Mexican-style cheese as a function of pH (5.0–6.5), NaCl (2–8% *w/w*) and moisture content (42–60%) which yields conservative predictions of pathogen behavior [100]. The same approach was applied to model the effects of NaCl (0–3% *w/w*), potassium sorbate (0–0.2% *w/w*), and nisin (0–240 ppm) in inhibiting the growth of *C. sporogenes* in a processed cheese analogue [97]. Moreover, logistic regression was applied to study the effects of NaCl (1.23–3.95%), sodium phosphate (1.31–3.35%), pH (5.4–6.2), and moisture (51–60%) on toxin production by *C. botulinum* in processed cheese [102].

More recently, Martinez-Rios et al. [5] developed a growth cardinal model which includes the parameter psi (ψ) that quantitatively indicates the distance between specific environmental conditions and the growth boundary of *L. monocytogenes* in spreadable processed cheese (Table 1). The ψ is estimated for a set of combinations of model variables. Where ψ is estimated to be lower than 1, it means that product characteristics and storage conditions place the pathogen on the growth side of the growth boundary, while on the no-growth side, ψ values are higher than 1 [112]. The mentioned model was further extended to include residual nisin as an explanatory variable of *L. monocytogenes* behavior in processed cheese, together with other environmental factors [96] (Table 1). These models aid in selecting product formulations and environmental conditions sufficiently distant to the growth boundary of the target pathogen, to assure that it does not grow in the evaluated cheese.

### 4.4. Microbial Interaction Models

Determination of pathogen growth or survival parameters in the presence of endogenous or intentionally added starter cultures in cheeses and the development of models considering their interactions are relevant to obtain more functional and realistic models.

Some effort was made to quantify and model the interaction of *L. monocytogenes* and LAB during ripening of Minas semi-hard cheese [103] and during storage of Cottage cheese [73] and Minas soft cheese [35]. In addition, Guillier et al. [104] investigated the mechanism of interaction between *L. monocytogenes* and biofilm microbiota present in wooden shelves used during the manufacture of a French smeared cheese. In general, in these studies the authors applied the Jameson effect or the Lotka–Volterra competition models or compared both approaches to depict the simultaneous growth of LAB and *L. monocytogenes*. In its simplest form, the Jameson-effect model assumes that LAB and *L. monocytogenes* inhibit each other to the same extent that they inhibit their own growth, and that one microorganism stops growing when the other has reached its maximum density [113]. To account for the growth inhibition caused by microbial competition, the Jameson model can be modified, including parameters describing the maximum critical concentration that a population should reach to inhibit the growth of the other population [114]. The Lotka–Volterra model assumes that the competition for a common substrate is described by two inhibition coefficients that must be estimated from the microbial growth curves in co-culture [115]. However, given the complexity and dynamic behavior of cheese microbiota, there is not much information on the development of validated microbial interaction models in the literature, and this could thus be a topic of further research.

### 4.5. Other Modeling Structures

In addition to primary and secondary models fitted using the classical two-step procedure or the one-step procedure, other modeling structures have been developed. Response surface methodology (RSM) has been applied to describe the influence of time (28 days) and temperature (4–15 °C) and their interaction on the *L. monocytogenes* population during the storage of a semi-soft cheese elaborated with raw-and pasteurized-milk cheese [8]. These authors found that both independent variables and their interaction exert a significant effect on *L. monocytogenes* population. In general, RSM allows to generate models with experimental designs consisting of a reduced number of experiments compared with factorial designs. Furthermore, the effects of the interactions between the independent variables on microbial populations can also be evaluated. On the other hand, the great disadvantage of RSM in the context of predictive microbiology is that the models derived are purely empirical and statistical and, therefore, have no biological meaning.

The use of neural networks (NN) to model the survival of *L. monocytogenes* in Katiki cheese during storage at temperatures from 5 to 20 °C has been investigated by Panagou, [81]. The developed model described *L. monocytogenes* survival equally well or slightly better compared with the classical Gompertz, Weibull and Geeraerd primary models. Validation at two temperatures within the model domain was performed successfully. NNs directly explore the relationship between the input variables and the output through a learning process until the network is trained on the presented data set. An NN does not restrict the type of relationship between input and output which means that, in contrast to conventional models, a mathematical equation must not be stated beforehand [81]. A potential practical advantage of the NN approach is that fitting and validation can be performed at the same time by the development of a single model. However, the extensive number of parameters and complexity of such models make them not fully suitable to be applied in all cases. Moreover, as with RSM, NN models do not include parameters with a biological meaning.

### 4.6. Computational Tools

Predictive models developed with microbial data obtained in cheeses have been implemented in software tools with user-friendly interfaces and can be used to obtain predictions of pathogen behavior in a quick and easy manner. The available software tools of foodborne pathogens on cheese matrices are represented in Table 3. Some of these applications, including ComBase Premium and MicroHibro [116] can be freely accessed online, while others can be downloaded and installed on users’ desktops such as Food Spoilage and Safety Predictor (FSSP) and GroPIN (Table 3). The FSSP encompasses an extensive validated model to predict the simultaneous growth of *L. monocytogenes* and LAB in Cottage cheese based on their cardinal parameter values [73]. The commercial software Dairy Products Safety Predictor can be used to perform simulations of an exposure or risk assessment model developed in dairy products [117].

The models available or to be implemented in MicroHibro can be further used to build QMRA models in the risk assessment module of the software. Other free web resources, such as ComBase [118] and Microbial Responses Viewer [119], include records of microbial responses in cheeses which can be applied in the development and validation of predictive models and in QMRA. These applications and databases have been constantly updated with the implementation of new peer-reviewed predictive models and data of microbial behavior and are relevant tools for decision-making to be used by food business operators, food safety researchers, or for didactic purposes.

### 4.7. General Considerations

The application of the predictive models available in software tools such as ComBase Predictor and the Pathogen Modeling Program to predict the microbial behavior in cheeses have often resulted in non-accurate estimates, with the overestimation of pathogen growth rates [105,106,120]. Models available in these software were developed in liquid laboratory media, which do not reflect the complex structure of cheeses [24,26]. Furthermore, the protective effect exerted by the technological microbiota of cheeses which limits the growth of pathogenic bacteria through competition and/or the production of bacteriocins has not been considered when developing the culture media models that are available in these software programs.

Realistic models must be able to handle the dynamic environment of the food matrix. In other words, models must reflect changes in pH, moisture, undissociated lactic acid concentration and other factors such as temperature that do not remain constant during cheese production and storage and play a relevant role in pathogen behavior [24]. To date, some predictive models have been generated under dynamic conditions in smeared-soft cheese [24,26,71] and Cottage cheese [73], reflecting the dynamic character of cheese production and storage. To enable a better understanding of the dynamics of the physicochemical parameters, coupling microbial models with the so-called technological models which describe changes in physicochemical parameters or the production of a metabolite throughout time might be a good strategy to obtain better-functioning and accurate predictive models. Examples of these technological models are those that relate the lactic acid concentration with its undissociated form [24,26,121], models that relate the a_w_ with the moisture content or weight loss [122] or models that relate the a_w_ with the NaCl concentration in the water phase salt [5].

Other investigations aimed at developing predictive growth models to be applied for different cheese types [123,124]. Augustin et al. [124] developed models to estimate the growth rates of *L. monocytogenes* in cheese based on growth data published in different studies and derived from challenge tests performed in many different types of cheeses, including Camembert, Brick, Brie, Cottage, and Queso Fresco. The variability in observed growth rates was very high and may have contributed to the poor performance of the models with regards to its accuracy (accuracy factor of approximately 3.5). The heterogeneity in pH, a_w_, lactic acid concentration and other unidentified abiotic or biotic factors such as differences in competitive microbiota between cheeses may be associated with the high variability in growth rates, although large variations in growth rates were also found in identical cheeses [124]. Their findings highlight the big limitation of developing a general predictive model for estimating the growth potential of a pathogen in different types of cheeses, which are complex and diverse food matrices.

Although several models are available in scientific literature describing microbial fate in cheeses, documentation on successfully validated models is limited. Martinez-Rios et al. [25] validated two models [5,73] out of nine published models to predict the growth of *L. monocytogenes* in mold/smear-ripened cheeses, with regards to the impact of temperature, pH, NaCl/a_w_, and lactic and acetic acids on growth rates. In addition, Centorotola et al. [125] validated a dynamic growth/death model originally designed to predict *L. monocytogenes* kinetics in a fermented meat product in Pecorino di Farindola cheese, elaborated with raw ewe’s milk. In both mentioned studies, the validated models could be used to evaluate the impact of storage conditions and cheese formulations on *L. monocytogenes* behavior. The use of predictive models represents a cheaper alternative to the performance of challenge tests [125]. However, it is essential to validate the models available in literature for a specific cheese type prior to their use, with regards to the specific characteristics and storage conditions of this product, due to the diversity and the variety of factors that simultaneously affect microbial behavior in cheeses.

## 5. Limitations and Future Challenges

Most of the fate studies performed so far were focused on *L. monocytogenes* behavior, which is undoubtedly the main hazard of concern in cheeses. However, more investigation on the fate of other foodborne pathogens such as *Salmonella* spp., *S. aureus*, STEC, and spore-forming bacteria, e.g., *Clostridium* spp., would be of great interest for cheese microbiological safety.

The high growth and survival capacity of foodborne pathogens on cheeses highlight the need for the development and optimization of control measures to increase the microbiological safety of these products, such as new biopreservation strategies and the application of non-thermal technologies.

On the other hand, although there are some published protocols reported elsewhere, a standardization of challenge testing experiments for microbial data generation should be considered in further studies to obtain quality data to be used for the development of predictive microbiology models.

Some effort might be made to develop predictive models capable of predicting both growth and inactivation on cheeses, since a shift from pathogen growth to inactivation is frequently observed due to changes in their physicochemical characteristics, production of lactic acid, and environmental conditions, along with production and storage.

Since cheeses are heterogenous matrices, more knowledge is needed regarding the effect of their physical structure on microbial behavior, especially regarding the biofilm forming ability of foodborne pathogens during cheese ripening and storage.

The interaction between the background microbiota or/and starter cultures and contaminating pathogens may be considered for model development in cheeses, together with the effects of processing parameters and product formulations.

Finally, the dynamic character of the cheese production process may be considered when developing predictive models, which would yield more realistic mathematical tools. The development of stochastic models describing microbial behavior in foods, considering the between- and within-batch variability in the physicochemical characteristics of cheeses would also result in more realistic estimates.

## 6. Conclusions

The main bacterial pathogens of interest concerning the safety of cheeses are *L. monocytogenes*, *Salmonella* spp., STEC, and *S. aureus*, besides spore-forming bacteria which have shown great survival and growth capacity in processed cheeses. The behavior of *L. monocytogenes* has been extensively evaluated in soft cheeses due to the occurrence of many listeriosis cases linked to cheese consumption. Regarding the main factors affecting microbial behavior in cheeses, pH, a_w_, undissociated lactic acid concentration, and temperature have been the most common explored in modelling studies, in addition to other organic acids and salts. Many modeling approaches have been developed to describe microbial growth and survival kinetics in cheeses during production and storage. Growth boundary models have also been developed to optimize cheese formulations and storage conditions. Cardinal models have shown great advantages compared with other secondary models applied to describe microbial growth rates in cheeses. The development of models with consideration to the dynamic character of cheese-making and ripening and microbial interactions between the technological microbiota of cheeses and contaminating pathogens result in more realistic estimates of microbial behavior. This review provides information that will assist food business operators and food safety researchers in the application of current available predictive models and in the design and optimization of cheese formulations, production processes and storage conditions to increase the microbiological safety of these RTE foods.

## Figures and Tables

**Table 1:** Microbial primary and secondary growth models developed in cheese.

Microorganism	Type According to Moisture (Cheese Name)	Description	Step Modeled ^1^	Primary Model ^2^	Secondary Model (Parameter Modeled)	Independent Variables (Ranges When Reported)	Reference
*Escherichia coli*	Soft ad spreadable (Mascarpone)	Non-ripened elaborated with pasteurized milk	Storage	Gompertz	Polynomial (Growth rate)	Time (9–21 days), temperature (3–15 °C)	Kowalik et al. [74]
	Soft (Brie)	Ripened elaborated with pasteurized milk	Storage	Baranyi	Ratkowsky (Growth rate) and linear (Lag)	Time (13 days), temperature (4–30 °C)	Kim et al. [33]
	Soft (Camembert)	Ripened elaborated with pasteurized milk	Storage	Baranyi	Ratkowsky (Growth rate) and linear (Lag)	Time (13 days), temperature (4–30 °C)	
	Processed (Mozzarella)	Non-ripened elaborated with pasteurized milk	Storage	Baranyi	Ratkowsky (Growth rate) and linear (Lag)	Time (13 days), temperature (4–30 °C)	
	Processed (Cheddar)	Non-ripened elaborated with pasteurized milk	Storage	Baranyi	Ratkowsky (Growth rate) and linear (Lag)	Time (13 days), temperature (4–30 °C)	
*Listeria monocytogenes*	Soft (Camembert)	Ripened elaborated with pasteurized milk	Storage	Baranyi	Polynomial (Growth rate)	Time temperature (3–15 °C)	Lobacz et al. [105]
	Soft (Blue)	Ripened elaborated with pasteurized milk	Storage	Baranyi	Polynomial (Growth rate)	Time temperature (3–15 °C)	
	Soft (Minas)	Ripened elaborated with raw/pasteurized milk, formulated with/without starter cultures	Storage	Baranyi	ND ^3^	Time (15 days) at 7 °C	Campagnollo et al. [67]
	Soft (Minas)	Ripened elaborated with raw/pasteurized milk, formulated without starter cultures	Production	Baranyi	ND	Time (22 days) at 22 °C	
	Semi-soft	Ripened elaborated with raw/pasteurized milk	Storage	Baranyi	ND	Time (0–28 days), temperature (4–15 °C)	Tiwari et al. [8]
	Processed and spreadable	Non-ripened elaborated with pasteurized milk	Storage	Logistic	Cardinal (Growth rate)	Time, temperature (3.8–22 °C), pH (6.1–6.6), a_w_ (0.952–0.975), lactic acid (6371–15,328 ppm), acetic acid (568–3483 ppm), citric acid (518–38,282 ppm), orthophosphate (0.14–4.98%), di-phosphate (<0.01–5.09%) and tri-phosphate (<0.01–5.17%), nisin (0.56–5.28 ppm)	Martínez-Ríos et al. [5,96]
	Soft and smeared	Ripened elaborated with raw and pasteurized milk	Production	Logistic	Cardinal (Growth rate)	Time, temperature, pH, a_w_, lactic acid	Schvartzman et al. [26]
	Soft or semi-soft and smeared	Ripened elaborated with raw milk	Production	Logistic	Cardinal (Growth rate)	Time (30 days)	Schvartzman et al. [70]
	Soft (Fresco)	Non-ripened elaborated with pasteurized milk	Storage	Gompertz	ND	Time at different constant temperatures (4 and 10 °C)	Leggett et al. [76]
	Soft blue white	Ripened elaborated with pasteurized milk	Storage	Logistic	Cardinal (Growth rate)	Time, temperature, pH, NaCl, undissociated lactic acid	Rosshaug et al. [24]
	Soft and smeared (Munster)	Ripened elaborated with pasteurized milk	Storage	Baranyi	Cardinal (Growth rate)	Time, temperature, pH, a_w_	Ferrier et al. [71]
	Soft (Fresco)	Non-ripened elaborated with pasteurized milk	Storage	Baranyi	Ratkowsky (Growth rate) and hyperbolic function (Lag)	Time (20 days), temperature (4–30 °C)	Thomas et al., [77]
	Soft (Fresco)	Non-ripened elaborated with pasteurized milk	Storage	Baranyi	Ratkowsky (Growth rate) and exponential (Lag)	Time (43 days), temperature (5–25 °C)	Uhlich et al. [78]
	Semi-hard (Coalho)	Ripened elaborated with pasteurized milk	Storage	Baranyi	ND	Time (14 days) at different temperatures (7.5 and 12 °C)	Araújo et al. [106]
	Soft (Minas)	Non-ripened elaborated with raw/pasteurized milk, formulated with/without starter cultures	Storage	Huang	Cardinal (Growth rate)	Time (15 days) at 7 °C, pH	Cadavez et al. [35]
	Soft (Minas)	Ripened elaborated with raw/pasteurized milk, formulated without starter cultures	Production	Huang	Cardinal (Growth rate)	Time (22 days) at 22 °C, pH	Gonzales-Barron et al. [85]
	Soft blue-veined (Gorgonzola)	Ripened elaborated with pasteurized milk	Storage	Baranyi	ND	Time (100 days) at 8 °C	Dalzini et al. [107]
	Soft (Cottage)	Non-ripened elaborated with pasteurized milk	Storage	Logistic	Cardinal (Growth rate)	Time, temperature (5–15 °C), pH (5.0–5.5), water phase salt (0–2%), lactic acid (0–2500 ppm in water phase), sorbic acid (0–1000 ppm in water phase)	Østergaard et al. [73]
	Soft and smeared	Ripened elaborated with pasteurized milk	Production	Baranyi	ND	Time	Guillier et al. [104]
*Staphylococcus aureus*	Semi-hard (Coalho)	Ripened elaborated with pasteurized milk	Storage	Baranyi	ND	Time (14 days) at different temperatures (7.5 and 12 °C)	Araújo et al. [106]
*Yersinia enterocolitica*	Soft (Camembert)	Ripened elaborated with pasteurized milk	Storage	Baranyi	Ratkowsky (Growth rate and Lag)	Time, temperature (3–15 °C)	Kowalik & Lobacz [62]

^1^ Production refers to both cheese-making and/or ripening. ^2^ In studies where more than one primary and secondary models have been fitted to data, only the ones presenting better fit and/or performance were reported in the table. ^3^ ND = Not developed.

**Table 2:** Microbial primary and secondary survival models developed in cheese.

Microorganism	Type According to Moisture (Cheese Name)	Description	Step Modeled ^1^	Primary Model ^2^	Secondary Model	Independent Variables (Ranges When Reported)	Reference
*Listeria monocytogenes*	Semi-hard (Minas)	Ripened elaborated with raw milk with/without starter cultures	Production	Log-linear	ND ^3^	Time (22 days) at 22 °C	Campagnollo et al. [67]
	Processed	Processed cheese derived from a hard cheese	Storage	Weibull	ND	Time at different temperatures (4–22 °C)	Angelidis et al. [80]
	Processed	Processed cheese derived from a hard cheese	Storage	Weibull	Arrhenius	Time, temperature (4–22 °C)	Angelidis et al. [82]
	Soft and spreadable (Katiki)	Non-ripened elaborated with pasteurized milk with the starter cultures	Storage	Geeraerd	Polynomial	Time, temperature (5–20 °C)	Mataragas et al. [79]
	Soft and spreadable (Katiki)	Non-ripened elaborated with pasteurized milk with the addition of starter cultures	Storage	Gompertz	Polynomial	Time (40 days), temperature (5–20 °C)	Panagou [81]
	Not specified	Non-ripened elaborated with pasteurized milk with the addition of starter cultures	Storage	Log-linear	ND	Time (15 days) at 4 °C	Sibanda & Buys [109]
	Not specified	Non-ripened grated cheese elaborated with pasteurized milk without the addition of starter cultures	Storage	Log-linear	ND	Time (120 days) at different temperatures (4 and 12 °C)	Valero et al. [36]
	Semi-hard	Ripened elaborated with raw milk with the addition of starter cultures	Storage	Weibull	Arrhenius and log-linear	Time (120 days), temperature (4–22 °C)	Valero et al. [84]
	Soft (Feta)	Ripened elaborated with pasteurized milk with the addition of starter cultures	Production	Log-linear	ND	Time (90 days) at 4 °C	Erkmen [111]
	Semi-hard (Minas)	Ripened elaborated with raw milk with and without the addition of starter cultures	Production	Log-linear	ND	Time (22 days) at 22 °C, pH	Gonzales-Barron et al. [85]
*Salmonella* spp.	Hard	Ripened elaborated with raw milk with the addition of starter cultures	Storage	Weibull	Arrhenius	Time (60 days), temperature (5–25 °C)	Lobacz et al. [75]
	Soft (Crottin)	Ripened elaborated with pasteurized milk with the addition of starter cultures	Storage	Churchil	ND	Time (42 days) at different temperatures (2–25 °C)	Tamagnini et al. [103]
*Yersinia enterocolitica*	Soft (Crottin)	Ripened elaborated with pasteurized milk with the addition of starter cultures	Storage	Vitalistic	ND	Time (42 days) at different temperatures (2–25 °C)	Tamagnini et al. [103]

^1^ Production refers to both cheese-making and/or ripening. ^2^ In studies where more than one primary and secondary models have been fitted to data, only the ones presenting better fit and/or performance were reported in the table. ^3^ ND = Not developed.

**Table 3:** Software tools integrating predictive models developed in cheese.

Software	Microorganisms	Cheese Type	Response	Model References	Software Available in
Food Spoilage and Safety Predictor (FSSP)	*L. monocytogenes*	Cottage cheese	Growth	Østergaard et al. [73]	http://fssp.food.dtu.dk/ (accessed on 5 January 2021)
Dairy Product Safety Predictor	*L. monocytogenes*, *Salmonella* spp., *S. aureus*, *E. coli*	Blue cheese, Cooked pressed Cheese, Soft cheese, Uncooked pressed cheese	Growth	-	www.aqr.maisondulait.fr (accessed on 5 January 2021)
GroPIN	Various	Various	Growth/Survival	-	www.aua.gr/psomas/gropin/ (accessed on 5 January 2021)
ComBase Premium	*E. coli*, *L. monocytogenes*	Camembert, Brie, Mozzarella, Cheddar, Semi-soft rind washed raw/pasteurized milk	Growth	Kowalik et al. [74], Kim et al. [33], Uhlich et al. [78], Tiwari et al. [8]	www.cbpremium.org (accessed on 5 January 2021)
MicroHibro	*L. monocytogenes*	Grated cheese, Semi-hard cheese	Survival	Valero et al. [84], Valero et al. [36]	www.microhibro.com (accessed on 5 January 2021)
